# Proteome Profile of Starch Granules Purified from Rice (*Oryza sativa*) Endosperm

**DOI:** 10.1371/journal.pone.0168467

**Published:** 2016-12-19

**Authors:** Shihai Xing, Xiaoxi Meng, Lihui Zhou, Hana Mujahid, Chunfang Zhao, Yadong Zhang, Cailin Wang, Zhaohua Peng

**Affiliations:** 1 Institute of Food Crops, Jiangsu Academy of Agricultural Sciences, Jiangsu High Quality Rice Research and Development Center, Nanjing Branch of China National Center for Rice Improvement, Nanjing, Jiangsu, China; 2 Department of Biochemistry, Molecular Biology, Entomology and Plant Pathology, Mississippi State University, Starkville, Mississippi, United States of America; USDA-ARS, UNITED STATES

## Abstract

Starch is the most important food energy source in cereals. Many of the known enzymes involved in starch biosynthesis are partially or entirely granule-associated in the endosperm. Studying the proteome of rice starch granules is critical for us to further understand the mechanisms underlying starch biosynthesis and packaging of starch granules in rice amyloplasts, consequently for the improvement of rice grain quality. In this article, we developed a protocol to purify starch granules from mature rice endosperm and verified the quality of purified starch granules by microscopy observations, I_2_ staining, and Western blot analyses. In addition, we found the phenol extraction method was superior to Tris-HCl buffer extraction method with respect to the efficiency in recovery of starch granule associated proteins. LC-MS/MS analysis showed identification of already known starch granule associated proteins with high confidence. Several proteins reported to be involved in starch synthesis in prior genetic studies in plants were also shown to be enriched with starch granules, either directly or indirectly, in our studies. In addition, our results suggested that a few additional candidate proteins may also be involved in starch synthesis. Furthermore, our results indicated that some starch synthesis pathway proteins are subject to protein acetylation modification. GO analysis and KEGG pathway enrichment analysis showed that the identified proteins were mainly located in plastids and involved in carbohydrate metabolism. This study substantially advances the understanding of the starch granule associated proteome in rice and post translational regulation of some starch granule associated proteins.

## Introduction

Rice (*Oryza sativa* L.) is one of the most vital crops in the world, which serves as the staple food for over half of the world’s population [1]. Rice endosperm is mainly composed of starch, a good source of carbohydrates [2]. The eating and cooking quality of rice-grain is directly connected to the starch composition in rice endosperm [3, 4]. There are two major types of starch in rice grains, highly structurally organized branched amylopectin and relatively unbranched amylose. The rice cooking quality and taste properties are primarily determined by the amylose/total starch ratio (amylose ratio or amylose content, AC) [5]. Rice varieties with high amylose content are cooked drier, with stiff and split up grain; while varieties with low amylose content are softer, with a more polished appearance, and sticky texture after cooking [6].

Starch, including both amylose and amylopectin, synthesized in chloroplasts is referred to as transient starch, while starch synthesized in amyloplasts is known as storage starch [7]. Located in starch granules in cereal endosperm, storage starch consists of glucose units combined by α-D-(1, 4)-glycosidic bond linkage producing linear chains, which have branch points introduced by α-D-(1, 6)-glycosidic bond linkages [7, 8]. The amylose molecules are essentially linear with less than 1% glucose units participating in α-(1, 6) bonds [7]. Differing from amylose, amylopectin is a branched polymer with roughly 5–6% branches [7]. One or more starch granule(s) can be packaged into an amyloplast [9].

Five classes of enzymes have been reported to be involved in starch biosynthesis. These enzymes consist of ADP-glucose pyrophosphorylase (AGPase), which produces the activated glucosyl donor ADP-glucose (ADPG) for starch synthesis; soluble starch synthase (SSS), which plays an important role in chain extension of starch; granule-bound starch synthase (GBSS), which is involved in both amylose and amylopectin biosynthesis in starch granule; starch branching enzyme (SBE) and starch debranching enzyme (DBE), which both influence the fine structure of amylopectin [10–15]. Each of these enzymes harbors several subunits or isoforms in plants [14]. AGPase has two isoforms, one isoform is a key cytosolic enzyme, while the other form plays a small enzymatic role in plastids [14–17]. AGPase is the first enzyme in the starch biosynthesis pathway that generates ADPglc, which is the sugar nucleotide used by starch synthesis enzymes in the amyloplast [14–16]. A portion of the produced ADPglc is then transported into the amyloplasts by the BRITTLE-1 (OsBT1) protein and used for starch synthesis [18, 19]. BRITTLE-1 (OsBT1) is a plastidial envelope protein functioning as an adenylate translocator [18, 19]. Two isoforms of GBSS have been found in rice: OsGBSS 1 located in the grain and OsGBSS 2 is mainly found in leaf [14, 20]. Given its exclusive location inside of the starch granule, some scientists consider GBSS as the most important enzyme in storage starch biosynthesis, especially in forming the extra-long chains of amylopectin [21]. SSS functions in extending the main oligosaccharide chains of amylopectin with the addition of hexose sequentially. Four isoforms of SSS have been reported, including SSS1, SSS2 (SSS2A /SSSII-3, SSS2B /SSSII-2, SSS2C/SSSII-1), SSS3 (SSS3A/SSSIII-2, SSS3B/SSSIII-1), and SSS4 (SSS4A/SSSIV-1, and SSS4B/ SSSIV-2) [14, 15]. Individual starch synthase isoforms can have unique roles [13] or overlapping functions in starch biosynthesis [22]. Both SBE and DBE are responsible for the fine structure of amylopectin [23], and there are four isoforms of SBE in rice grain including OsSBE1, OsSBE3 (OsQEIIa, or OsBEIIb), and OsSBE4 (OsQEIIb, or OsBEIIa) [14, 24, 25], while two types of DBE isoforms including isoamylase (OsISO) and pullulanase (OsPUL) have been identified in rice. ISO has at least three isoforms (OsISO1, OsISO2, and OsISO3), but no isoforms of PUL has been reported in rice [14, 26]. Many of these enzymes are allocated amongst the soluble fraction of the plastids and the insoluble starch granules in rice endosperm [7, 27]. Some of these enzymes might physically associate [13] and assemble into functional complexes [28].

Starch in rice endosperm is mainly synthesized in the amyloplast by a series of enzymes [10–16, 19–26]. Except for some soluble enzymes existing in the amyloplast stroma [16, 29, 30], most enzymes involved in starch biosynthesis are entirely or partially starch granule-associated and starch is deposited in a well-organized structure with layers of crystalline lamella and amorphous lamella arranged alternatively [7, 8, 31–34]. Given that senescence immediately follows starch synthesis in rice seeds and the unique structure of starch granule prevents other enzymes from freely entering and leaving the starch granule [35, 36], it is for good reason to hypothesize that many starch synthesis related proteins are entrapped in the matured starch granules. Identification of these proteins would provide insight into mechanisms of starch synthesis, regulation of rice grain quality and the structure of starch granules. Unfortunately, little is known about the starch granule proteome in rice thus far although several studies have been published in maize, wheat, barley and other plants [37–39]. To better understand all the proteins associated with the starch granule and the packaging of starch granule components, we developed a protocol for rice starch granule purification from mature endosperm, examined the starch granule proteome using LC-MS/MS, and compared the proteomes extracted by two different protein extraction methods. The results provided a reliable method for starch granule purification and novel insight into starch granule proteome composition as well as post translational modification of some starch granule associated proteins.

## Results and Discussion

### Starch granule purification from mature rice endosperm and examination

**Fig 1:**
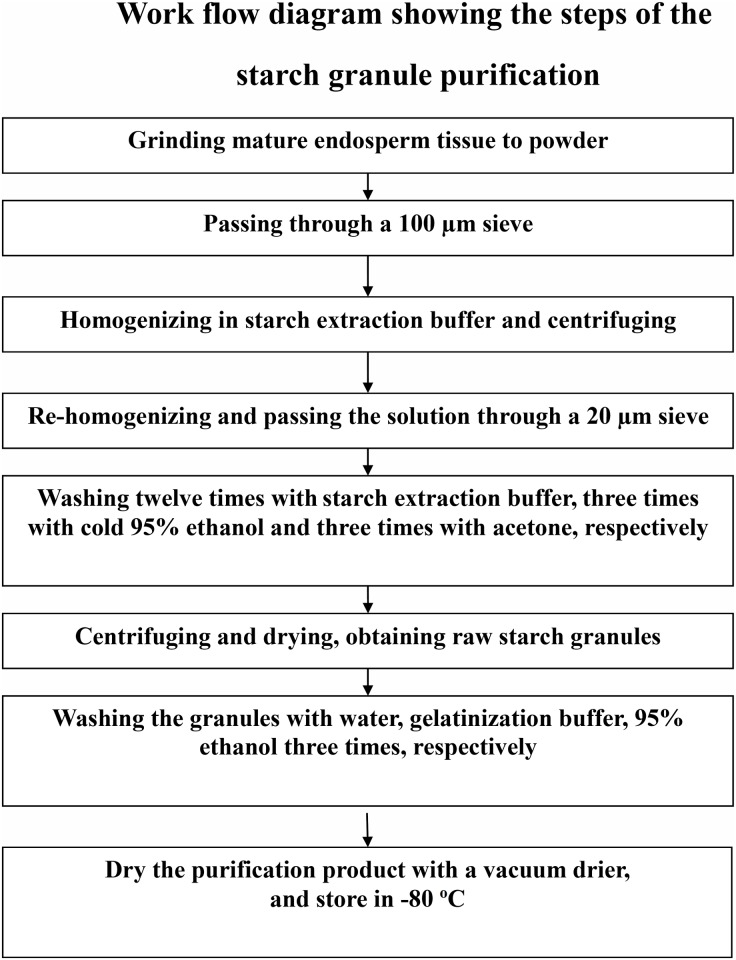
Work flow diagram showing the steps of the starch granule purification. Mature rice endosperm were used as the starting materials for this experiment. The final products were the purified starch granules used for further experiments in this report.

To study the starch granule proteome of rice, we developed a protocol to purify starch granules from mature rice endosperm with reference to other cereal’s starch granule purification protocols [7, 38, 40]. The general procedure used for rice starch granule extraction is shown in a work flow diagram (Fig 1). With this method, highly purified starch granules were obtained in a large scale.

**Fig 2:**
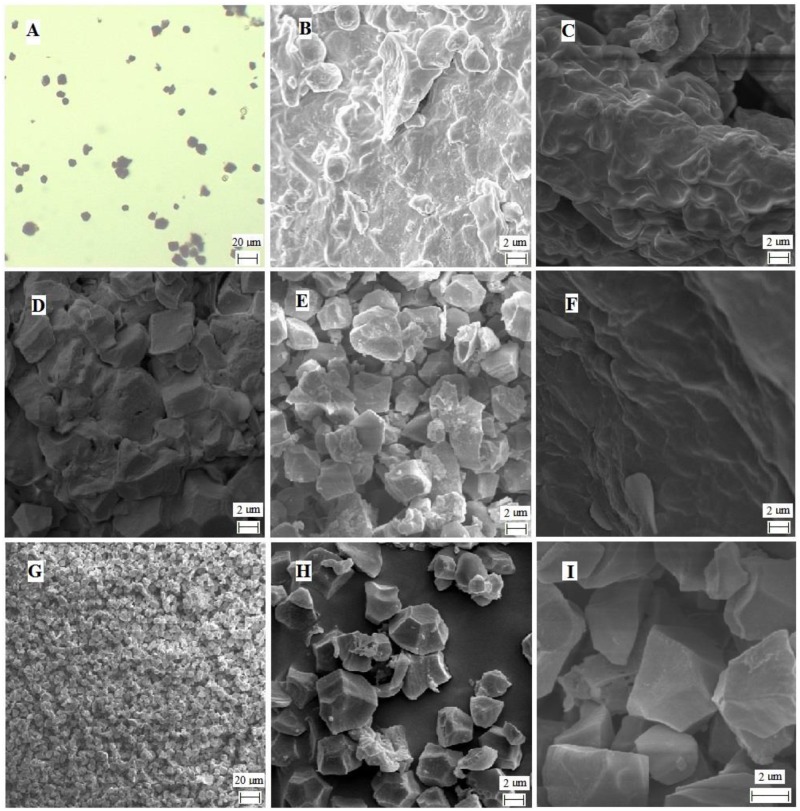
Image of purified starch granules and the intermediate products of purification. A: I_2_ stain image of the purified starch granules. Microscope observation with 40 × amplification; B: Cross section image of rice endosperm viewed by SEM. 6k amplification; C: Large endosperm fragment image under SEM. 6k amplification; D: Endosperm fragment image under SEM. 6k amplification; E: Partially purified starch granule image under SEM, 6k amplification; F: The sediments of grounded endosperm image under SEM, 6k amplification; G-I: Purified starch granule image at different magnifications under SEM. G: 1 k amplification; H: 6k amplification; I: 24 k amplification.

To examine the quality of the purified starch granules, the resulting starch granules were stained by I_2_ and examined under a microscope (Fig 2A). The blue color indicated that the purified particles could be stained by I_2_ and the estimated size of the purified particles was about 10 μm in diameter, which is consistent with the reported starch granule size [41].

To further evaluate the purity of the starch granules that we obtained, SEM observation was carried out. The rice endosperm fragments, the intermediate products of the starch granule purification steps, and the purified starch granules were monitored (Fig 2B–2I). As shown in Fig 2, individual starch granules could hardly be seen in intact endosperm (B), large endosperm fragments (C and D), and the large sediments of spin washing (F). While, an individual starch granule can be seen in partially purified starch granules (E), and purified starch granules (G, H, and I). In addition, the crystal structure is very clear under SEM. Given that the purified starch granules were well stained by I_2_ (Fig 2A), the results suggested that our starch granule preparation was of good quality.

### SDS-PAGE and Western blot analyses of purified rice starch granule proteome

**Fig 3:**
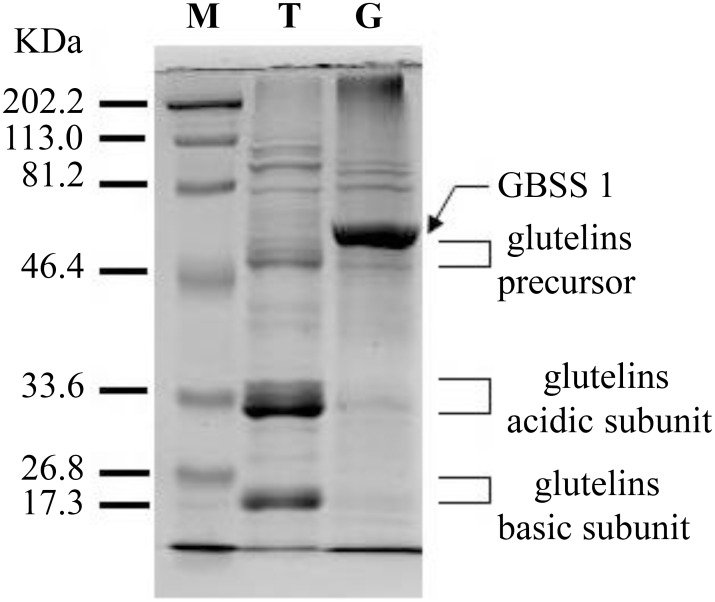
The pattern of starch granule proteins and total proteins of rice endosperm on SDS PAGE. The proteins extracted from rice endosperm and rice starch granules using phenol extraction method were separated by 12% SDS-PAGE and visualized by Coomassie blue stain. M: Marker; T: Total proteins; G: Starch granule proteins.

To examine and identify the starch granule proteome, we extracted the proteins using phenol extraction method as reported previously [42, 43] and compared the starch granule proteome with the rice endosperm total proteome on a SDS-PAGE. As shown in Fig 3, glutelins were the predominant proteins of rice endosperm total proteome as we reported before [44]. The most abundant protein (the thickest band) in the purified starch granule proteome was not visible in the total protein lane, suggesting a good enrichment of the protein during purification. This thick band, verified by LC-MS/MS identification, was GBSS I (granule-bound starch synthase I) (Fig 3), which is consistent with GBSS I being the major component in starch granules [10, 45–47], Meanwhile, the most abundant proteins (the glutelins) in the endosperm total proteome became barely visible in the purified starch granule proteome. These results demonstrated that glutelins were mostly removed and starch granule associated proteins were effectively enriched. Given that the storage proteins are highly abundant in the seeds, however, it is impossible to completely remove the storage proteins during purification unless highly specific affinity purification steps are used.

**Fig 4:**
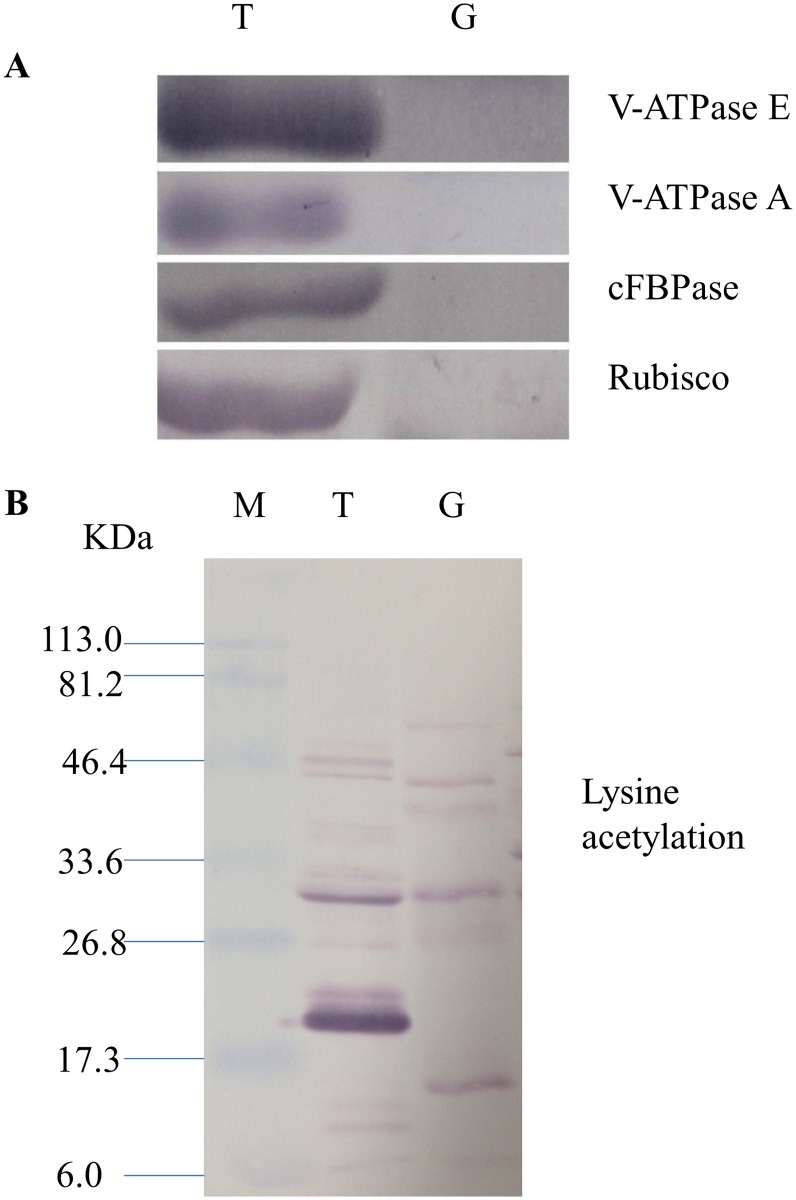
Western blot image of the starch granule proteome. Same amount of proteins (25 μg per lane) were loaded. A: Western blot images with different antibodies. The anti-bodies used were for V-ATPase E, V-ATPase A, Anti-Rubisco, cFBPase (Agrisera, Sweden). T: Total proteins; G: Starch granule proteins. B: Western blot image of protein acetylation of endosperm and starch granule proteins. Antibodies for acetylated Lysine (ImmuneChem) were used for Western blots. The source of proteins is indicated on the top of the lane. M: Protein marker; T: Total proteins extracted from endosperm; G: Proteins extracted from starch granules.

To further validate the effectiveness of starch granule purification, we carried out Western blot analysis with antibodies specific for known cytosolic and organelle proteins. The Western blot results showed that plant vacuolar proteins VHA-E and VHA-A were detected in the rice endosperm total proteins but were not detected in the protein preparation of purified starch granules (Fig 4A). Similarly, cytosolic protein cFBPase and chloroplast protein Rubisco were also detected in the rice endosperm total protein but not in the protein preparation of purified starch granules (Fig 4A). These results indicated that the cytosolic and other organelle proteins were effectively removed during purification.

### LC-MS/MS analysis of the starch granule proteome

To identify the proteins associated with starch granules, we extracted the proteins from the enriched starch granules by both phenol extraction method [42, 43] and Tris-HCl buffer extraction methods [45] with three replicates followed by protein identification with LC-MS/MS.

The proteins identified by LC-MS/MS are listed in S1 and S2 Tables (each of the sheets shows one replicate in the table), respectively. Mass analyses of all the replicates identified 695 proteins in the samples extracted by Tris-HCl buffer (listed under A in Table 1 and the details are provided in S3 Table) and 1157 proteins in the samples extracted by phenol buffer (listed under B in Table 1 and the details are provided in S3 Table), respectively. Since the proteome extracted by the phenol buffer contained more proteins, covering almost all the proteins identified in the sample extracted by Tris-HCl buffer, the phenol extraction method appears to be a better method for starch granule proteome recovery. In the following analysis, we will mainly focus on the proteins identified with the phenol extraction method.

**Table 1:** GO Distribution of the Starch Granule Proteins.

Items			A	B
Identify Proteins			695	1157
GO Analysis	Biological Process	metabolic process	366	683
		cellular process	329	606
		response to stimulus	227	366
		single-organism process	155	260
		localization	81	127
		developmental process	57	95
		biological regulation	54	97
		multicellular organismal process	51	86
		cellular component organization or biogenesis	41	84
		multi-organism process	36	66
		other	58	103
	Cellular Component	cell	429	764
		organelle	347	610
		membrane	268	440
		macromolecular complex	155	271
		membrane-enclosed lumen	89	127
		extracellular region	59	89
		other	3	5
	Molecular Function	binding	335	589
		catalytic activity	251	523
		structural molecule activity	83	123
		transporter activity	31	52
		nutrient reservoir activity	19	26
		enzyme regulator activity	16	25
		other	28	90
Subcellular Localization		extracellular	43	72
		mitochondria	50	75
		chloroplast	240	407
		nuclear	97	130
		cytoskeleton	11	18
		nuclear	2	2
		endoplasmic reticulum	8	20
		vacuolar membrane	13	22
		plasma membrane	33	51
		chloroplast, mitochondria	1	2
		peroxisome	3	9
		cytosol	194	349

The probability of a protein being detected in mass analysis is positively proportional to its abundance in the sample when the protein size is normalized [48–50]. The peptide count is used to measure protein abundance by counting the number of times a peptide was identified for a given protein [48–50]. Therefore, comparison of peptide counts of the proteins being identified in the sample is informative in determining protein abundance. We ranked all the identified proteins in order by their peptide counts in S3 Table. S3 Table shows that the known starch granule associated protein GBSS1 ranked highest on the list of identified proteins, with a peptide count of 2769 after MS/MS analysis in the phenol extraction sample. In addition, most of the other known starch granule associated proteins were also identified with peptide counts over 100 and mostly ranked in the upper portion of the S3 Table. The results suggested that we had a highly in depth analysis of the starch granule proteome using LC-MS/MS approach and our purification worked well. The known starch synthesis related proteins are summarized in Table 2 with peptide counts listed, namely GBSS 1 (Q0DEV5, peptide count 2769), SBE3 (Q6H6P8, peptide count 472), SBE1 (Q0D9D0, peptide count 448), SSS1 (Q0DEC8, peptide count 386), pullulanase (Q7X834, peptide count 269), Pho 1 (Q9AUV8, peptide count 260), AGPS (P15280, peptide count 206), AGPLar (Q5VNT5, peptide count 193), SSII-3 (Q0DDE3, peptide count 179), GBSSII (Q8GTK0, peptide count 103), SSII-1 (Q7XE48, peptide count 63), DULL1 (Q6Z1D6, peptide count 21), AGPLar3 (Q6AVT2, peptide count 16), putative DBE, ISO2 (Q6AU80, peptide count 8). Given that the protein size difference among these proteins is within a few folds, the peptide count numbers can roughly reveal the protein quantity.

**Table 2:** Starch Synthesis and Related Proteins Identified in Starch Granules Proteome.

*UniProtKB*	Counts	Protein names	Gene names
Q0DEV5	2769	Granule-bound starch synthase 1 (GBSS 1)	WAXY, Os06g0133000
Q6H6P8	472	Starch Branching enzyme-3 (SBE 3)	SBE3, Os02g0528200
Q0D9D0	448	Starch Branching enzyme-1 (SBE1)	SBE1, Os06g0726400
Q0DEC8	386	Soluble starch synthase 1 (SSS I)	Os06g0160700
Q7X834	269	pullulanase (PUL), plastidial ADP-glucose transporter	OSJNBa0019G23.2
Q9AUV8	260	Alpha-1,4 glucan phosphorylase (SP) (Pho1)	OSJNBa0040E01.3
P15280	206	Glucose-1-phosphate adenylyltransferase small subunit (AGPS)	AGPS Os08g0345800, Os09g0298200
Q5VNT5	193	Glucose-1-phosphate adenylyltransferase large chain (AGPLar)	P0663E10.9
Q0DDE3	179	Soluble starch synthase 2–3 (SSII-3)	SSII-3,Os06g0229800
Q8GTK0	103	Starch synthase (GBSSII)	P0710F09.134 or GBSSII,Os07g0412100
Q7XE48	63	Soluble starch synthase 2–1 (SSII-1)	SSII-1,Os10g0437600
Q6Z1D6	21	Putative starch synthase DULL1	OSJNBa0056O06.4–1
Q6AVT2	16	Glucose-1-phosphate adenylyltransferase (AGPLar 3)	OSJNBa0027J18.8, Os03g0735000
Q6AU80	8	Putative isoamylase-type starch debranching enzyme ISO 2 (DBE)	OSJNBa0014C03.3
Q6YZC3	4	Glucose-6-phosphate/phosphate translocator	B1099H05.2, P0020B10.26

In addition to the well-known starch granule associated proteins listed above, we also identified a few other candidate proteins for involvement in starch synthesis related functions. Hsp70 (Q2QV45) has been considered to play a role in protein folding in amyloplast stroma and maintaining enzyme activity in starch granule in maize endosperm [51]. Therefore, it is probable to consider it as an essential component of the starch granule. Interestingly, amyloplastic Hsp70 was detected with a peptide count of 205. Furthermore, putative Brittle-1 protein (Q6Z782), a transporter of phosphor-glucose that is critical for starch synthesis based on genetic studies [19], was identified with a peptide count of 259 in the starch granule associated proteome, suggesting that this protein may also function as a starch granule protein as well.

Other candidates for possible starch synthesis related functions included alpha-1, 4 glucan phosphorylase (Q9AUV8) (Pho 1) with a peptide count of 260 and sucrose synthase (P30298) also referred to as sucrose-UDP glucosyltransferase with a peptide count of 187. Alpha-1, 4 glucan phosphorylase functions through releasing alpha-D-glucose 1-phosphate by using phosphate to break alpha 1, 4 bond linkages between pairs of glucose residues at the end of long glucose polymers [52]. Genetic studies by Satoh et al. (2008) have shown that mutation of plastidial alpha-glucan phosphorylase gene in rice has effect on the biosynthesis and structure of starch in endosperm [53]. Identification of this protein in the starch granule proteome purification further suggests that this enzyme may be starch granule associated.

Sucrose synthase has been shown to display both synthetic activity and degradative activity in maize endosperm [54]. Its specific role in granule starch synthesis and its association with starch granule is not known thus far. Our observation suggests sucrose synthase (P30298) may have direct interaction with the starch granule and it is worthwhile to further explore whether this enzyme is related to starch biosynthesis.

Moreover, Q0DA62, a glycoside hydrolase family member, was identified with a peptide count of 164. Since this enzyme is probably involved in the breakdown of amylose and/or amylopectin and other degradation enzymes have been shown to be critical for starch synthesis [55], it will be interesting to further study if this enzyme is involved in starch synthesis in the endosperm. Another possible starch granule associated protein included protein Q6ZBH2 with a peptide count of 153. This protein belongs to the alcohol dehydrogenase superfamily (zinc-type) and maybe involved in fructose biosynthesis [56]. Its connection with starch synthesis is still not clear.

Additionally, Pyruvate, phosphate dikinase 1 (Q6AVA8, PPDK) was identified with a peptide count of 291. PPDK functions in catalyzing the conversion of a pyruvate to phosphoenolpyruvate (PEP) [57]. In the process, this reaction uses up 1 molecule of ATP and makes one molecule of AMP [55, 57]. A protein similar to PPDK in function, phosphoglucan water dikinase (EC 2.7.9.5) has also been proposed to play a role during starch degradation [58]. Due to the high abundance of PPDK in the starch granule associated proteome, it will be interesting to further test if PPDK also plays any role in starch synthesis in rice.

In depth analysis of the proteome introduces the possibility of uncovering many contaminating proteins, even if the contaminating proteins are in lesser abundance. Here, the most abundant contaminating proteins identified were the storage proteins known as glutelins. Comparison of summary peptide counts of identified glutelins with GBSS1 protein showed glutelins were identified with roughly 30% or less of the peptide count of the GBSS1 protein. Glutelins are the most abundant storage proteins in the rice endosperm proteome. Before starch granule purification, GBSS1 was not detected on SDS page (see Fig 3, lane T), the fact that fewer peptides of glutelins were detected in LC-MS/MS analysis when compared with GBSS1 is consistent with our observation on SDS-PAGE that GBSS1 was substantially enriched and glutelins were removed during starch purification (Fig 3, lane G). Given that glutelins are contaminating proteins during purification, they were not included in further analysis. Other detected seed storage proteins included cupin family protein (Q75GX9 and Q852L2), allergenic protein (Q8H4M4), and allergen RAG2 (Q0D7S4).

**Fig 5:**
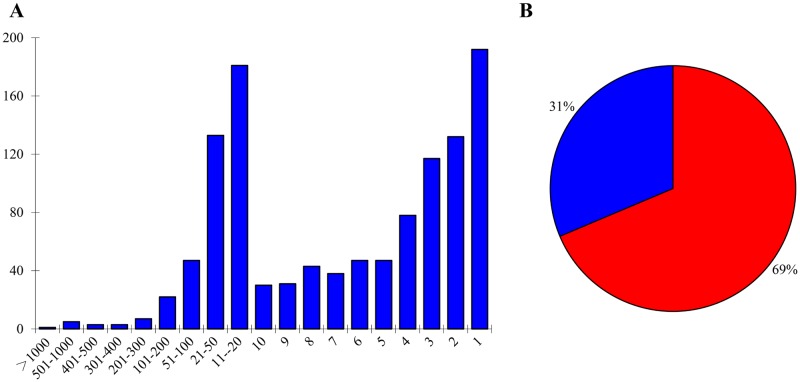
Distribution of identified proteins based on their peptide counts. A: Protein peptide count numbers vs numbers of proteins. The proteins identified with the same peptide counts were grouped together to obtain the protein numbers. X-axis: protein peptide counts; Y-axis: number of proteins. B: Peptide count percentage distribution of 40 proteins with highest peptide counts (excluding storage proteins) in the chloroplast/amyloplast (red) and other organelles (blue).

The diagram in Fig 5 examines the protein distribution versus the protein peptide counts (Fig 5A). Among the 1157 identified proteins, 91 proteins were identified with a peptide count of 50 or more (S3 Table). And 1066 identified proteins were identified with a peptide count of less than 50 (S3 Table), which is less than roughly 1.9% of the peptide count of the GBSS1 protein, suggesting that these proteins were in a low abundance. Additionally, 720 proteins were identified with <10 peptide count, which is less than roughly 0.37% of the peptide count of the GBSS1 protein. These proteins were either in extremely low abundance in the starch granule or they were contaminating proteins with very low abundance in the sample.

### GO and KEGG pathway analysis

To better understand the proteins associated with starch granules, we carried out gene ontology (GO) analysis. The results showed that 683 (59.03%, 683/1157) phenol extracted proteins were associated with metabolic processes (Table 1, S4 Table). Protein subcellular localization analysis showed that chloroplast/amyloplast was the dominant category of cellular localization (S5 Table), which is consistent with the subcellular distribution nature of starch granules. 69% of the identified peptide counts from the 40 proteins with highest peptide counts (excluding storage proteins) were located in the chloroplast/amyloplast, suggesting that the enriched proteins were mainly from starch granules (Fig 5B and S6 Table).

**Fig 6:**
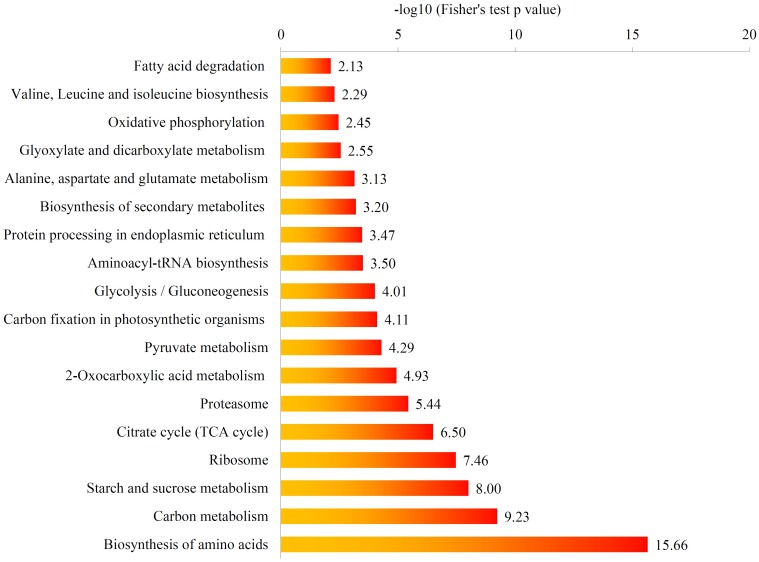
KEGG pathways enriched in the starch granule proteome. KEGG pathway enrichment analysis of starch granule proteome. The value of -log10 (Fisher's test p value) is shown.

**Fig 7:**
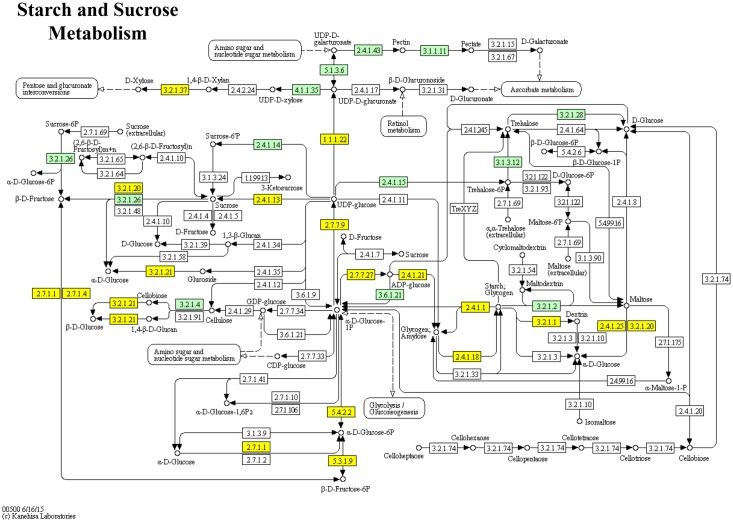
Proteins enriched in starch and sucrose metabolic pathways. The enzymes marked with yellow and blue color are proteins enriched in rice starch granule proteome.

We further investigated the enriched pathways in which starch granule associated proteins were involved by using Kyoto Encyclopedia of Genes and Genomes (KEGG) database. Functional Annotation Tool of DAVID against the background of rice (*Oryza sativa* L.) protein database was specifically employed. The result showed that the starch and sucrose metabolism pathway were significantly enriched in the purified starch granule proteome (Figs 6 and 7). These proteins included 15 in starch and sucrose metabolic pathways, 13 in glycolysis and gluconeogenesis pathway, 7 in citrate cycle (TCA cycle) pathway, and 28 in carbon metabolism pathway, respectively (S7 Table). These metabolic pathways are all related to carbohydrate biosynthesis or catabolism.

### Protein modification in starch granule proteome

Many reports have shown that metabolic pathway enzymes are subject to extensive posttranslational modifications such as acetylation [59–62]. In this study, we achieved significant coverage of the starch granule associated proteins with deep mass analysis, including GBSS 1, with up to 85.7% coverage of its protein sequence (S2 Table). Taking advantage of this, we searched various modifications against identified peptides based on a mass shift in the corresponding residue induced by the modifications. We found lysine acetylation modifications in multiple key enzymes of the starch synthesis pathway, including modifications of P15280 (AGPS), Q0D9D0 (SBE 1), Q0DEV5 (GBSS 1), Q5VNT5 (AGPLar), Q6H6P8 (SBE 3), Q9AUV8 (Pho 1), (S8 Table and mass peak shown in S1 Fig). Protein modification by lysine acetylation in starch granules was further verified by western blot with anti-lysine acetylation antibody (Fig 4B). The functional significance of these modifications on these proteins requires further investigation. It has been reported that some of the starch synthesis enzymes and their isoforms assembled into functional complexes [28], and the formation of these complexes maybe regulated by protein modifications such as protein phosphorylation [13, 28]. It is worthwhile to investigate the function of lysine acetylation on enzymes which are involved in starch synthesis in regulating complex assembly and enzyme functions.

## Conclusions

We have developed a protocol to effectively purify starch granules from mature endosperm of rice. The enrichment of the starch granules was verified by light and SEM microscopy, I_2_ staining, and Western blot examinations. We found that phenol extraction method is highly effective in the recovery of starch granule associated proteins. Mass analysis of the purified starch granules proteomes identified the previously known starch granule associated proteins, as well as several candidate proteins for possible functional involvement in starch synthesis. Moreover, lysine acetylation was identified on multiple starch synthesis pathway proteins, indicating a possible role of this modification in regulation of starch synthesis.

## Materials and Methods

### Plant materials and growth conditions

Rice (*Oryza sativa* L., Nipponbare) plants were grown at 28°C during day and 23°C at night in the greenhouse of the Department of Biochemistry and Molecular Biology, Mississippi State University, MS, USA. The top five spikelets were labeled and harvested after maturation. Collected seeds were dried and incubated for 15 h at 40°C in 0.3% (W/V) sodium metabisulphite and 85% (v/v) lactic acid (pH 3.8) to inactivate proteases during rehydration [7]. Endosperm tissues were manually dissected from pericarps and embryo tissues.

### Starch granule extraction

Starch granules from mature rice endosperm were purified with a method modified from the reported protocol developed in maize [7]. Endosperm tissue was ground and passed through a 100 μm mesh size sieve. The resulting fine powders were mixed with starch extraction buffer (50 mM Tris-HCl pH 7, 10% glycerol, 10 mM EDTA, and 1.25 mM DTT) at 4°C. The homogenate was centrifuged at 15,000 rpm for 15 min at 4°C, and the viscous layer which was on the top of the starch was removed carefully. The precipitate was re-homogenized in the starch extraction buffer and passed through a 20 μm mesh size sieve. The starch pellet was washed twelve times with starch extraction buffer, three times with cold 95% ethanol, and three times with acetone, each wash was followed by centrifugation at 8000 rpm for 10 min at 4°C, and then the pellet was dried under a speed vacuum drier (model LYPH-LOCK 6, LABCONCO). At this point of the procedure, the starch granules were considered as crude because they still contained contaminating proteins attached to their surface. To obtain more purified granules, the crude granules were washed three times in water, three times in gelatinization buffer (62.5 mM Tris-HCl, pH 6.8; 2% SDS, and 5% β-mercapto ethanol), and three times in 95% ethanol, then lyophilized under a vacuum drier. The dried starch granules were stored in -80°C for optical observation and protein extraction.

### Microscopic and SEM observation

The purity of the extracted starch granules was monitored by staining with I_2_ solution at a concentration of 0.08 mg/100ml and observed under the microscope. For SEM (Scanning Electron Microscope) examination, the dried pellets from different stages during starch granule extraction were dusted on the surface of a carbon-adhesive tab and sputter-coated with 45nm platinum particles using EMS 150-T (Electron Microscopy Sciences) [63, 64]. SEM examination of starch granules and the intermediates from purification steps was performed using a Zeiss EVO-50 scanning electron microscope at 10.0 kV [63, 64].

### Protein extraction

Two methods were used to extract proteins from starch granules, which are the Tris-HCl extraction method and Phenol extraction method.

#### Tris-HCl method for protein extraction from starch granules

The process of protein extraction from rice starch granules by Tris-HCl method was as follows: starch granules were minced into powder, Tris-HCl buffer (50mM Tris-HCl pH 8.8, 5 mM EDTA, 20 mM DTT, 100Mm KCl, and 2mM PMSF added freshly) was added, boiled for 5 min, and then homogenized for more than 30 minutes at 4°C by vortex. The homogenate was centrifuged at 10 000rpm for 10 minutes at 4°C, and the supernatant was collected. The extraction was repeated three times by adding Tris-HCl buffer, vortex and then centrifugation. The final collected supernatant was mixed with five volumes of 100% acetone to precipitate the proteins at -20°C, incubating for more than 2 h or overnight. The precipitant was obtained by centrifugation at 10, 000 rpm for 15 minutes at 4°C and the pellets were washed more than four times with cold 80% acetone. The protein pellets were lyophilized in a speed vacuum and stored at -80°C for further analysis [42].

#### Phenol method for protein extraction from starch granules

Proteins of starch granules were extracted by phenol extraction method as reported with some modifications [42, 43]. Briefly, the materials were mixed with a protein extraction buffer (0.9 M sucrose, 0.5 M Tris-HCl pH 8.7, 0.05 M EDTA, 0.1 M KCl, 1% Triton X-100 and 2% β-mercaptoethanol added freshly), boiled for 5 min to release the granule proteins and inactivate the protease. Equal volume of saturated phenol (pH 8.0) was added and then homogenized for more than 30 minutes at 4°C. The homogenate was centrifuged at 7000 rpm for 15 minutes at 4°C, the phenol phase was collected. The phenol extraction was repeated three times by adding protein extraction buffer, vortexing and centrifugation. The final collection of phenol phase was mixed with five volumes of precipitation buffer (methanol with 0.1 M ammonium acetate and 1% β-mercaptoethanol added freshly), vortexed and stored at -20°C overnight. The precipitant was collected by centrifugation at 12000 rpm for 15 minutes at 4°C and the pellets were washed three times with cold precipitation buffer followed by three times with ice cold 70% ethanol. The protein pellets were lyophilized in a speed vacuum and stored at -80°C for further analysis [42].

### SDS PAGE analysis

For SDS-PAGE, proteins extracted by phenol method from rice endosperm and starch granules were separated on 12.0% SDS PAGE gel and were stained with Coomassie Brilliant Blue (CBB) for protein visualization.

### Western blot analysis

Proteins were separated on a 12% SDS-PAGE gel and electro-transferred onto a PVDF membrane (Millipore) for Western blots. The membrane was treated with a block solution (5% m/V non-fat milk, 0.05% v/v tween-20, and 1 X TBS) overnight at 4°C. After blocking, the membrane was incubated with corresponding primary antibody for 2 hours at room temperature, followed by incubation with respective quantity of alkaline phosphatase conjugated secondary antibody for 90 minutes. After three 10 min washes, signal detection was carried out using NBT/BCIP (nitroblue tetrazolium/5-bromo-4-chloro-3-indolyl phosphate) detection system.

### Protein identification by LC-MS/MS and bioinformatics analysis

#### Trypsin digestion

Trypsin digestion procedure was the same as previously reported [42, 43] with minor modifications. The proteins extracted by Tris-HCl method and phenol methods, with three replicates each, were re-dissolved in urea buffer (8 M urea, 100 mM TEAB (tetraethylammonium bromide), pH 8.0) and the protein concentration was determined with a 2-D Quant kit (GE Healthcare Life Sciences) according to the manufacturer’s instructions. Prior to protein digestion, proteins were reduced with 10 mM DTT (for 1 h at 37°C) and alkylated with 20 mM IAA for 45 min at room temperature in the dark. Before the addition of trypsin, the protein solution was diluted by adding 100 mM TEAB to reduce urea concentration to less than 2 M. Then, the sequencing grade trypsin (Promega Corporation) was added at a 1: 50 (w/w) trypsin-to-protein mass ratio for digestion overnight. In order to make certain of complete protein digestion, 1: 100 (w/w) trypsin-to-protein mass ratio was added for another 4 h digestion at 37°C. After digestion was completed, peptides were desalted by a Strata X C18 SPE column (Phenomenex), followed by vacuum drying.

#### LC-MS/MS analysis

Desalted peptides were dissolved in 0.1% FA (Formic acid) and 2% ACN (Acetonitrile) (Solvent A), directly loaded onto a reversed-phase pre-column (Acclaim PepMap 100, Thermo Scientific). Peptides separation with a reversed-phase analytical column (Acclaim PepMap RSLC, Thermo Scientific) at a constant flow rate of 300 nl/min with a 70 min linear gradient from 5 to 25% solvent B (0.1% FA in 98% ACN) was performed, followed by an increase from 25 to 35% solvent B for 12 min, further climbing to 80% in 4 min holding at 80% for the last 4 min on an EASY-nLC 1000 UPLC system.

After peptide separation, the resulting peptides were analyzed by Q Exactive hybrid quadrupole-Orbitrap Plus mass spectrometer (ThermoFisher Scientific). Peptides were subjected to a NanoSpray Ionization (NSI) source followed by tandem mass spectrometry in Q Exactive (Thermo Fisher Scientific) coupled online to the UPLC. A resolution of 70,000 was used to detect intact peptides with the Orbitrap. For peptide selection, 28% NCE was used for MS/MS and a resolution of 17,500 was used to detect ion fragments in the Orbitrap. A procedure which was data dependent was used, alternating between one MS scan followed by 20 MS/MS scans was used for the topmost 20 precursor ions above a threshold ion count of 1×10^4^ in the MS survey scan with 30.0s dynamic exclusion. A 2.0 kV electrospray voltage was applied, as well as automatic gain control (AGC) to overfilling of the ion trap; 5×10^4^ ions were accrued for generation of the MS/MS spectra. The m/z scan range was 350 to 1800Da for MS scans.

#### Database search

The subsequent MS/MS raw data was processed by using Mascot search engine (*version 3*.*2*) [65]. Tandem mass spectra were searched against the *UniProt*_*Oryza sativa* (63,195 sequences) concatenated with reverse decoy database. Two missing cleavages were allowed for the trypsin/P (indicated as the cleavage enzyme) per peptide. The mass error was set to 10 ppm and 0.02 Da for precursor ions and fragment ions, respectively. Acetylation on the protein N-terminal was found from the mass peak of the peptides. The peptide ion score was set to >20.

#### Protein annotation and subcellular localization

The UniProt-GOA database (http://www.ebi.ac.uk/GOA/) was used to obtain the gene ontology (GO) annotation for identified proteins. Protein IDs were first converted into a UniProt ID, followed by mapping to GO identifications. If an identified protein was not annotated by the UniProt-GOA database, a protein sequence alignment method using InterProScan software was used to annotate the protein’s gene ontology functions. Gene Ontology annotation was based on the following three categories: biological process (BP), cellular component (CC) and molecular function (MF).

Prediction of the subcellular localization of identified proteins was achieved by using Wolfpsort, which is an updated version of PSORT/PSORT II for the prediction of the subcellular localization of eukaryotic proteins.

#### KEGG pathway and enrichment analyses

Kyoto Encyclopedia of Genes and Genomes (KEGG) database was used to identify enriched pathways. The annotation result was mapped on the KEGG pathway database using KEGG online service tool KEGG mapper. Moreover, enriched pathways were identified using the KEGG database by the Functional Annotation Tool of DAVID against the background of *Oryza sativa*.

To test for enrichment of proteins in a specific category and pathway enrichment analyses, a two-tailed Fisher’s exact test was conducted which tested the enrichment of the differentially expressed proteins against all identified proteins. Correction for multiple hypothesis testing was performed utilizing standard false discovery rate (FDR) control methods. A corrected p-value < 0.05 was considered significant for gene annotation and those pathways were classified into hierarchical categories according to the KEGG website.

## Supporting Information

S1 Fig Mass peak of Kac modified peptides in starch biosynthesis pathway. (PDF)

S1 Table Tris-HCl method_protein list (A1, A2, and A3). (XLS)

S2 Table Phenol method_protein list (B1, B2, and B3). (XLS)

S3 Table Tris-HCl method, Phenol method_protein annotation summary (A and B). (XLS)

S4 Table Protein_GO-Terms_Level2_Classify. (XLSX)

S5 Table Proteins subcellular localization. (XLS)

S6 Table The ratio of peptide counts distribution between chloroplast/amyloplast and other organelle proteins for proteins with the highest peptide counts (subtracting storage proteins). (XLS)

S7 Table Proteins enriched in carbohydrates pathways (starch and sucrose metabolism, Citrate cycle pathway, Glycolysis and gluconeogenesis and Carbon metabolism). (XLS)

S8 Table Proteins with lysine acetylation modification in starch synthesis pathway. (XLS)
