# Supplementary material for: Proteome Profile of Starch Granules Purified from Rice (Oryza sativa) Endosperm
Source: PLoS One. 2016 Dec 19;11(12):e0168467. doi: 10.1371/journal.pone.0168467 (PMC5167393; doi:10.1371/journal.pone.0168467)

Mass Peak of Peptides Had been Modified by Kac in Starch and Sucrose Biosynthesis Pathway

P15280-217:

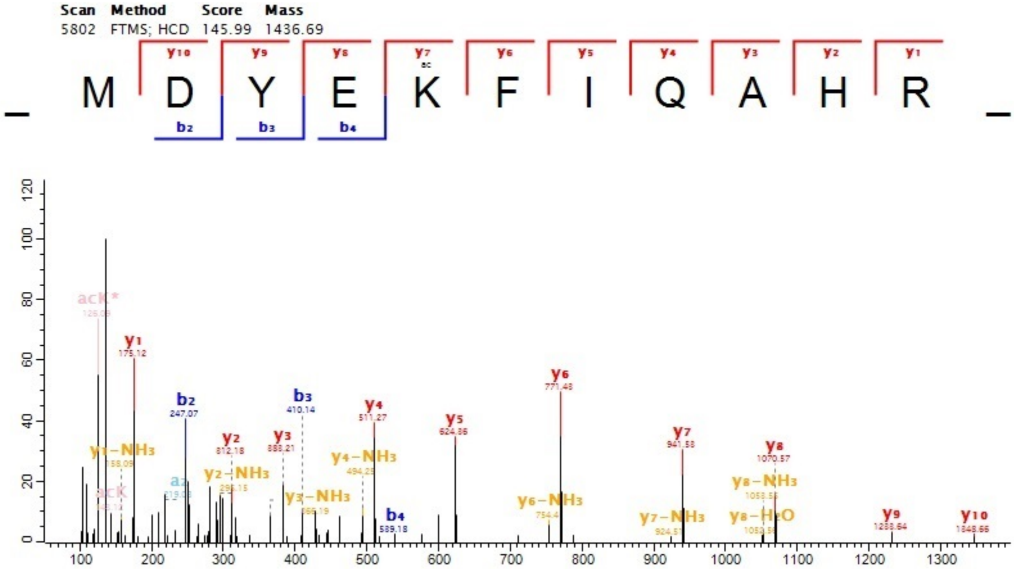


P15280-261:

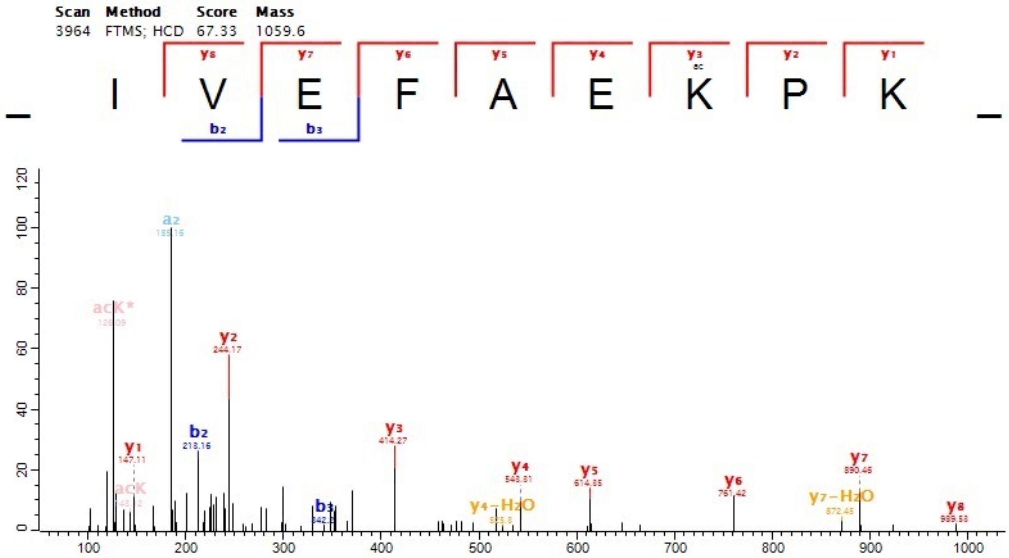


Q0D9D0-89:

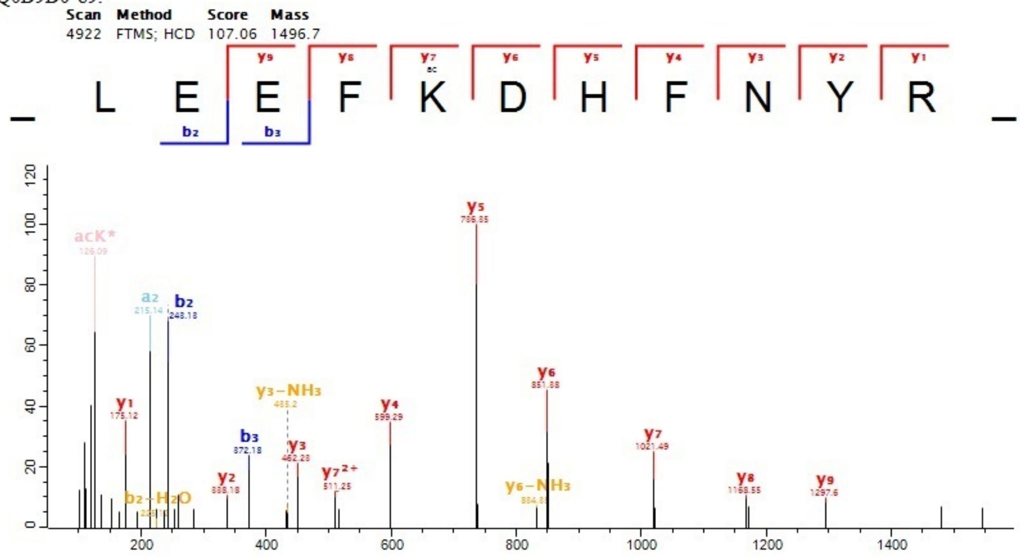

Q0D9D0-103:

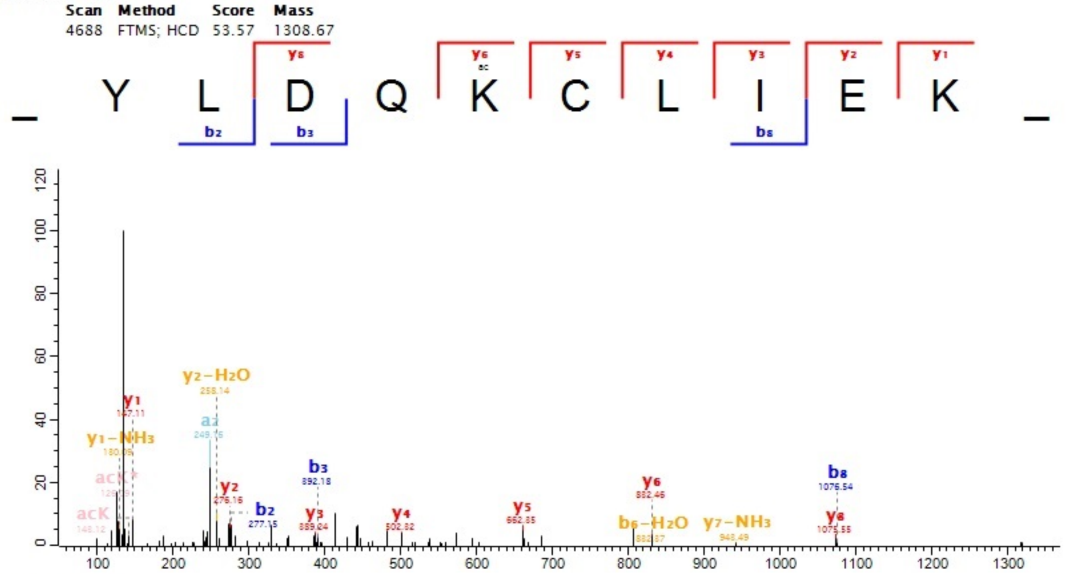


Q0D9D0-118:

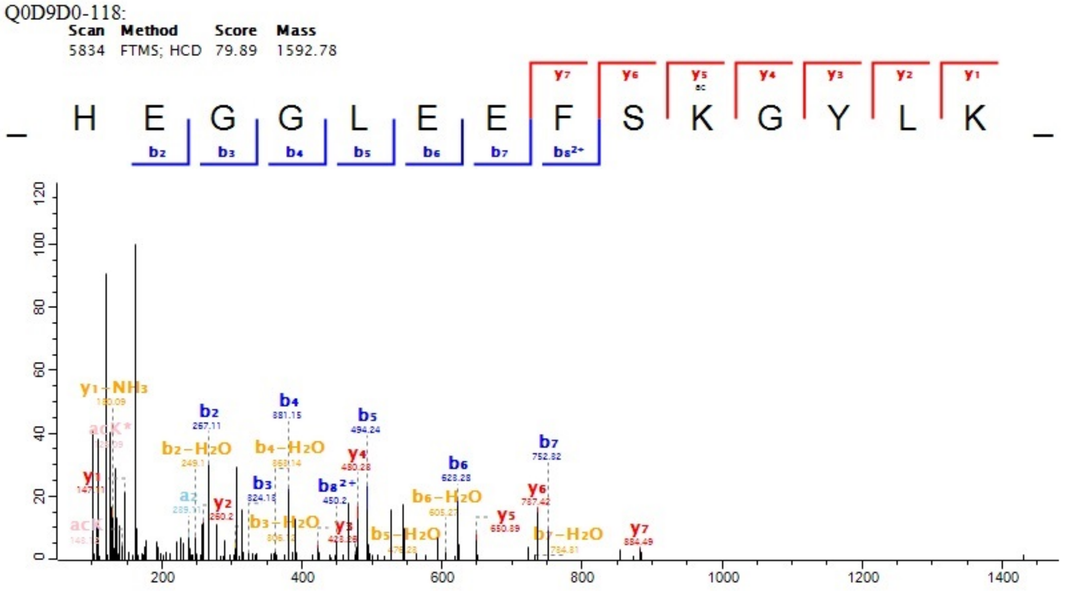


Q0D9D0-164:

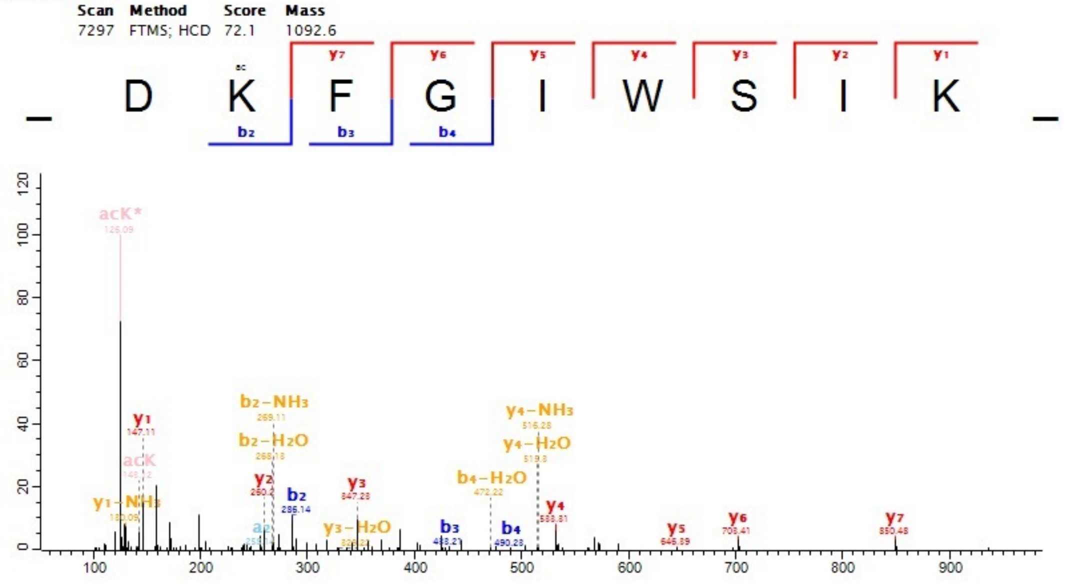

Q0D9D0-236:

| Scan | Method | Score | Mass |
|---|---|---|---|
| 2607 | FTMS; HCD | 121.56 | 1803.99 |

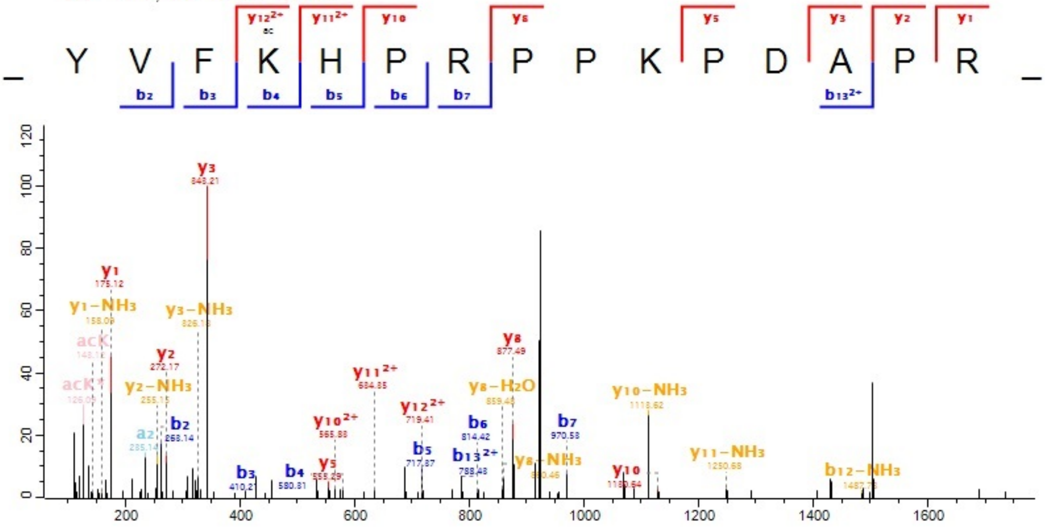


Q0D9D0-372:

| Scan | Method | Score | Mass |
|---|---|---|---|
| 3809 | FTMS; HCD | 99.01 | 1160.57 |

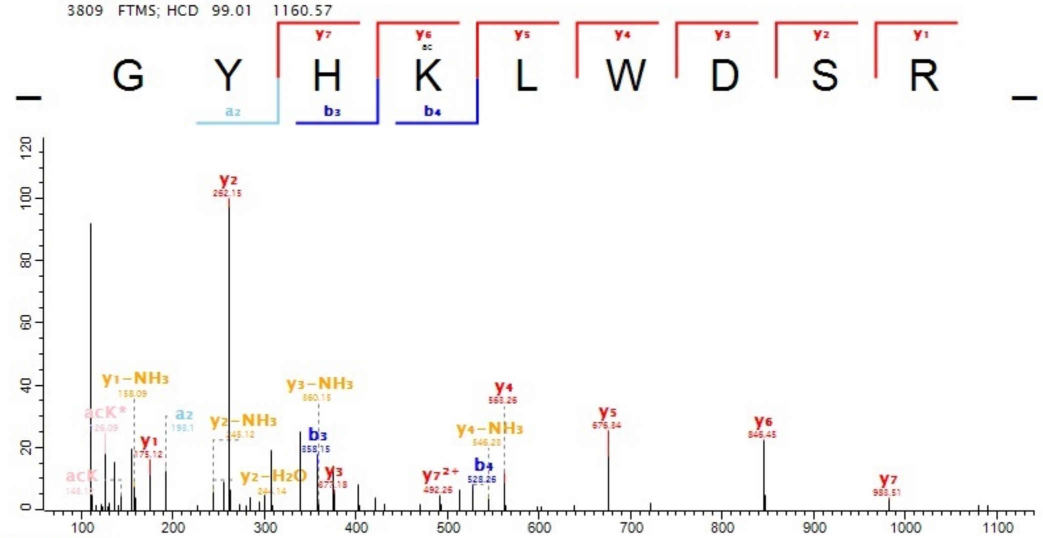


Q0D9D0-614:

| Scan | Method | Score | Mass |
|---|---|---|---|
| 3380 | FTMS; HCD | 112.94 | 1427.59 |

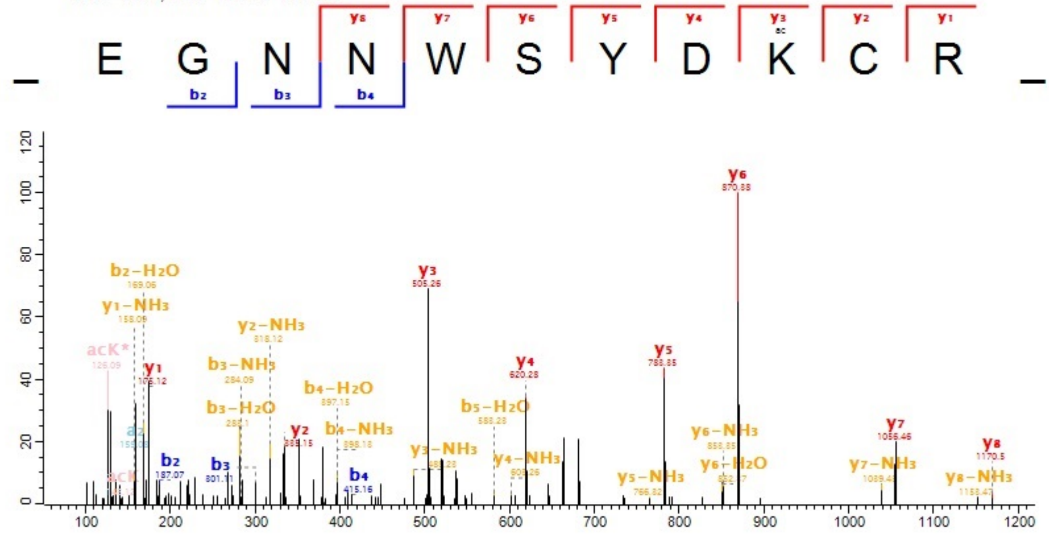

Q0D9D0-662:

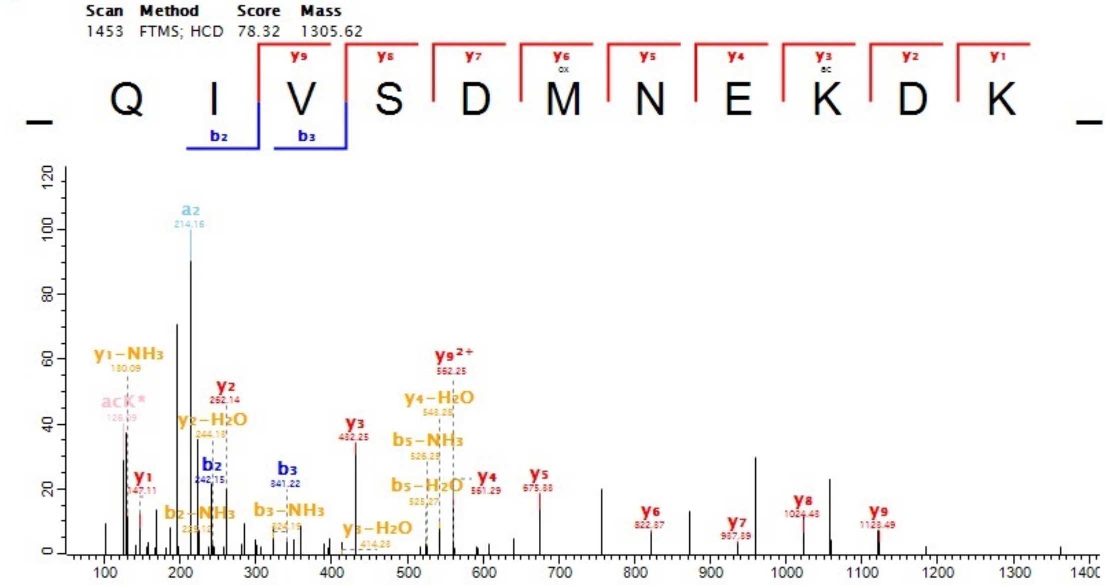


Q0D9D0-697:

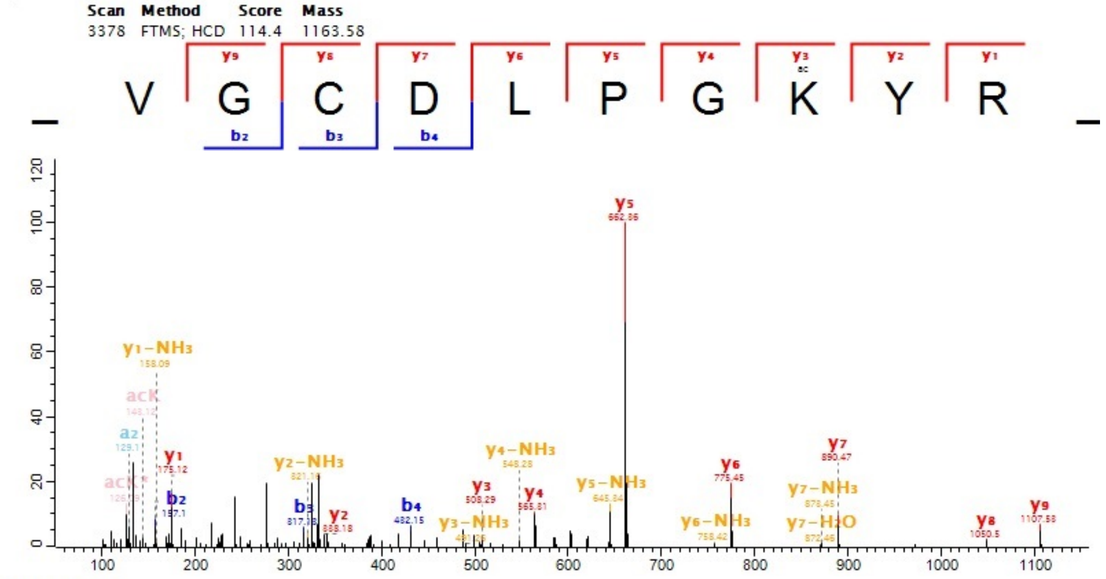


Q0D9D0-809:

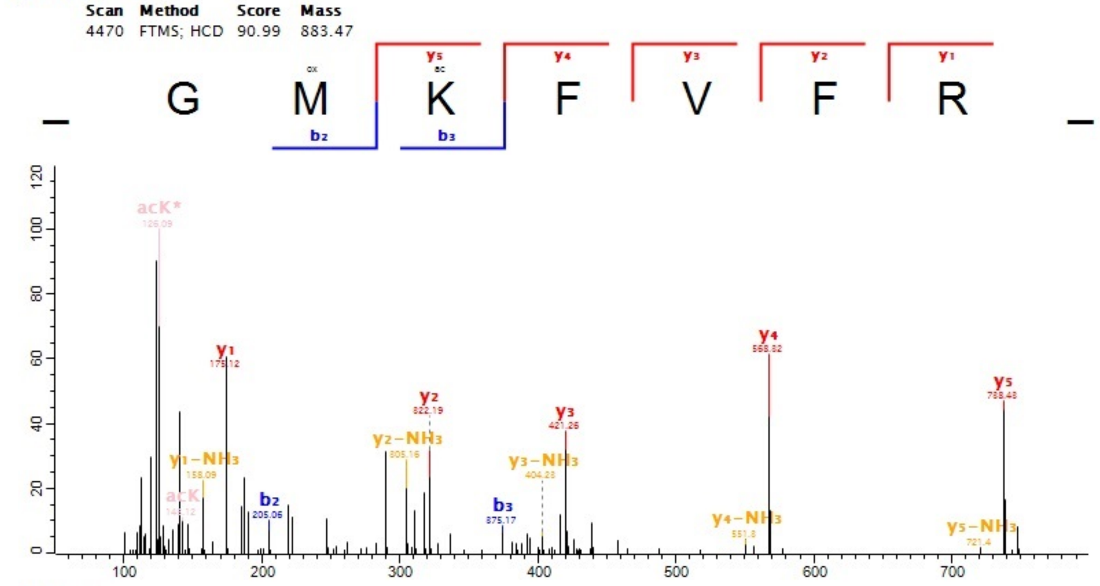

Q0DEV5-444:

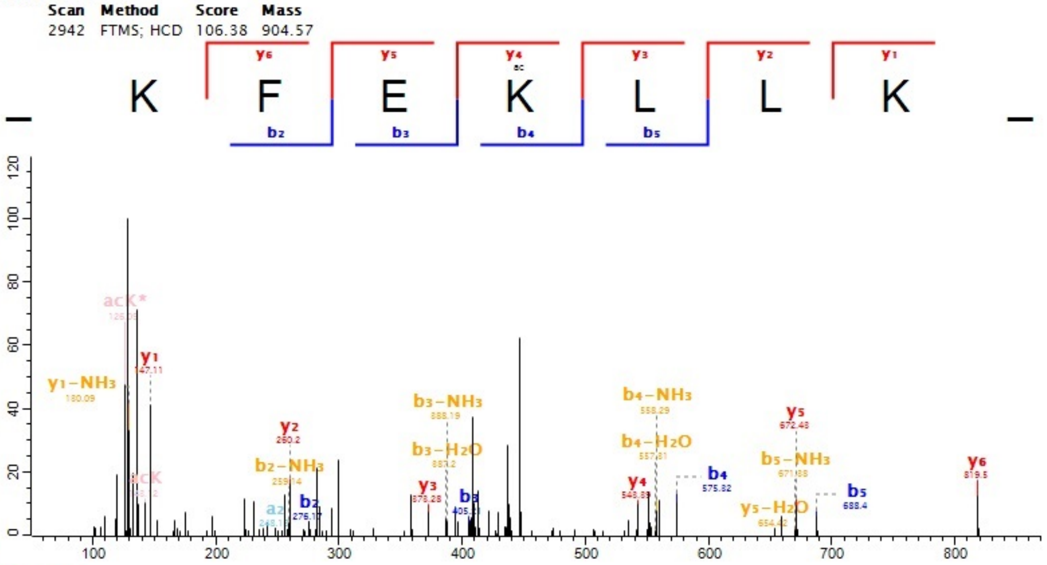


Q0DEV-452:

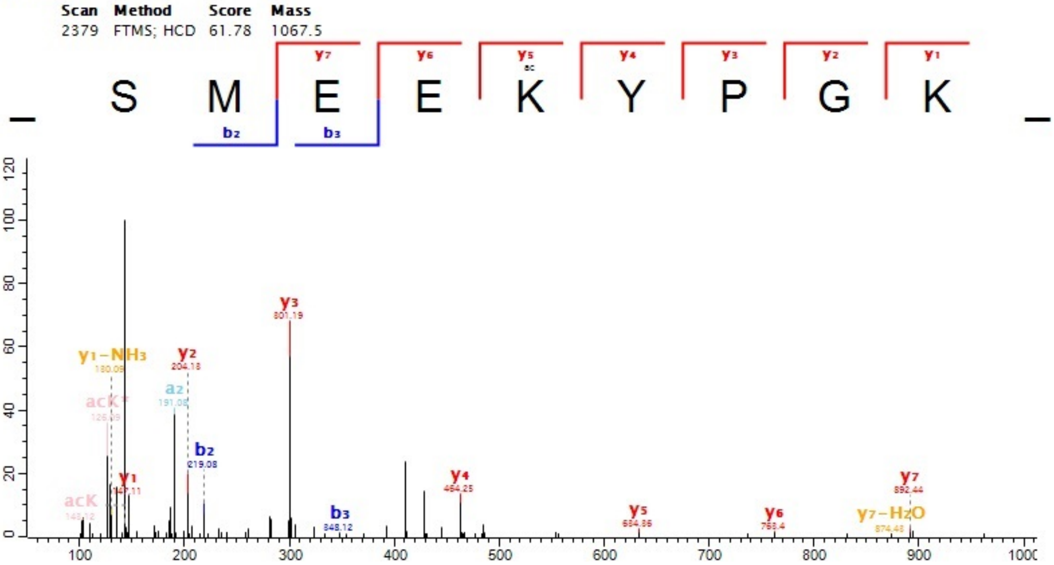


Q5VNT5-250:

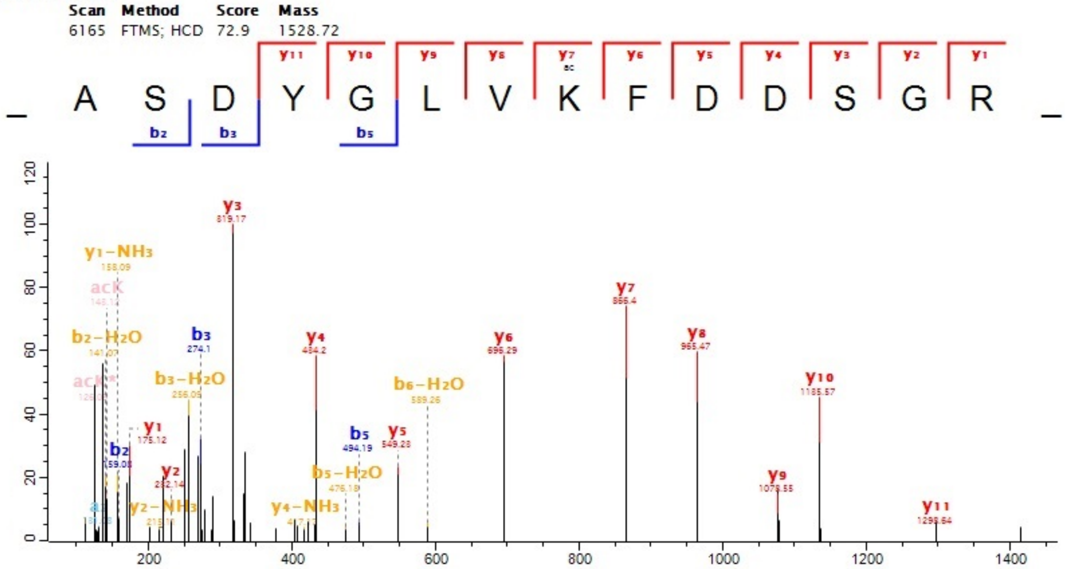

Q5VNT5-260:

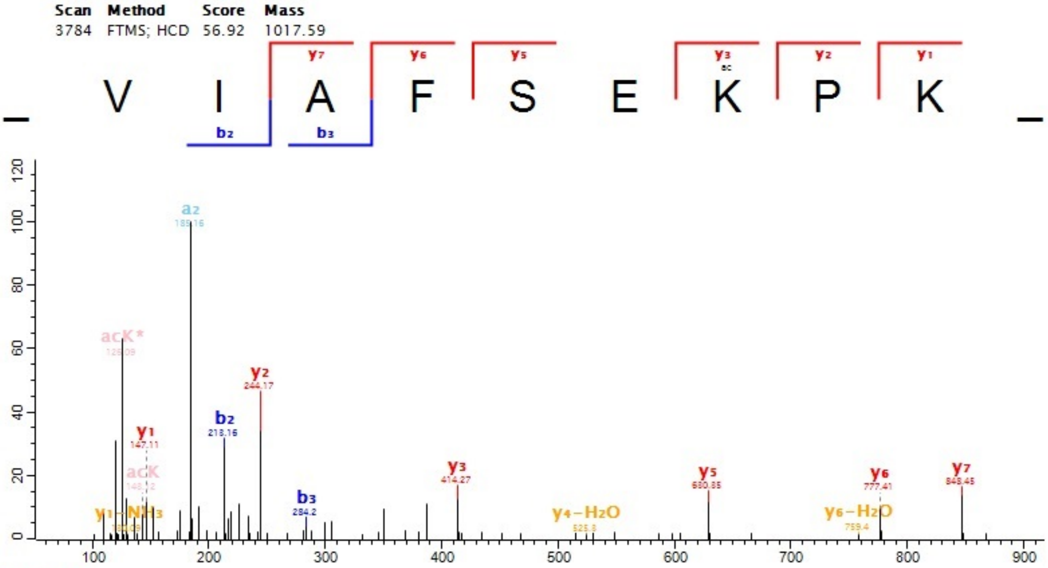


Q6H6P8-738:

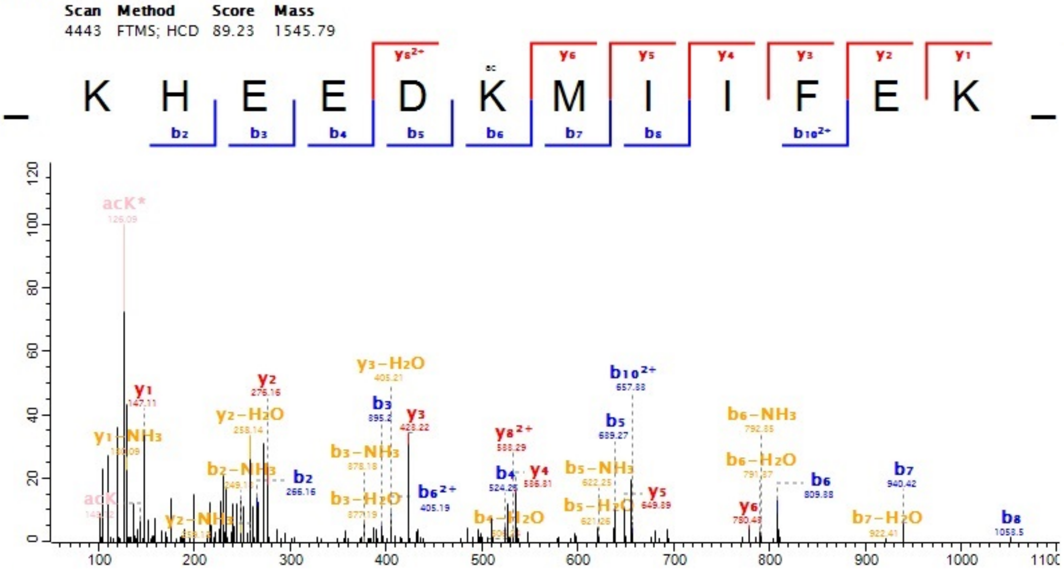


Q6H6P8-771:

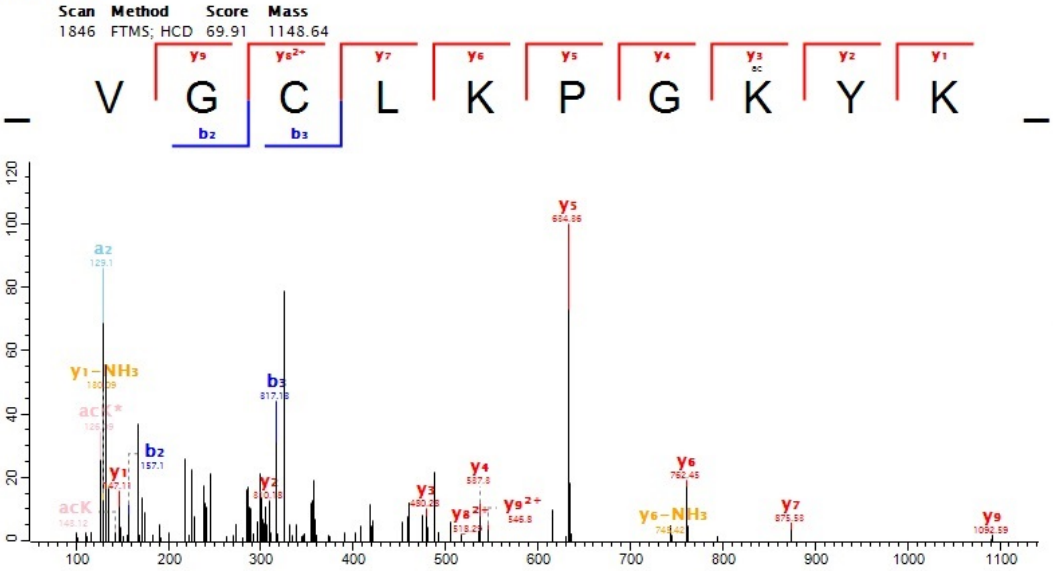

Q9AUV8-216:

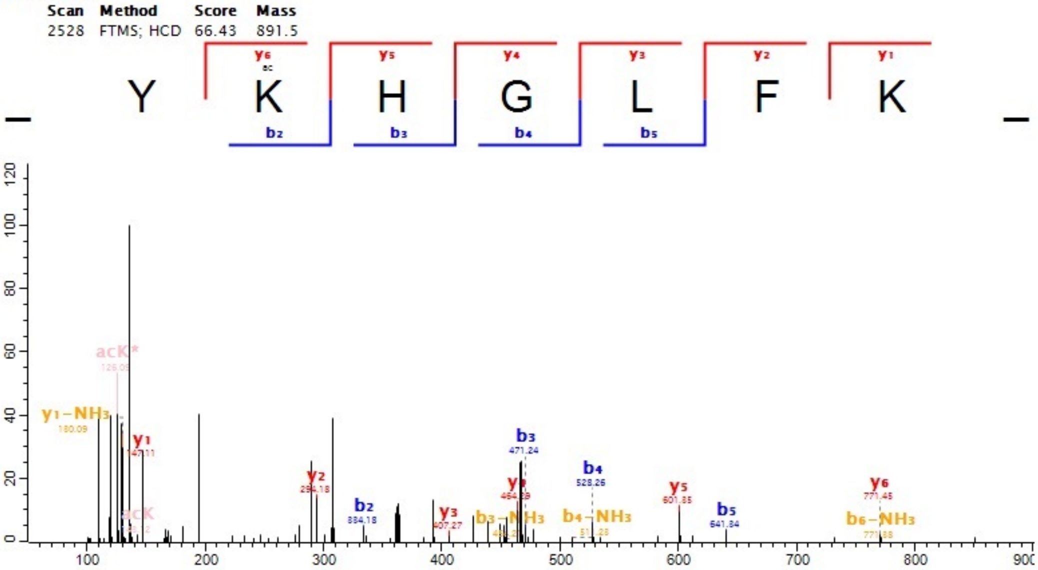


Q9AUV8-255:

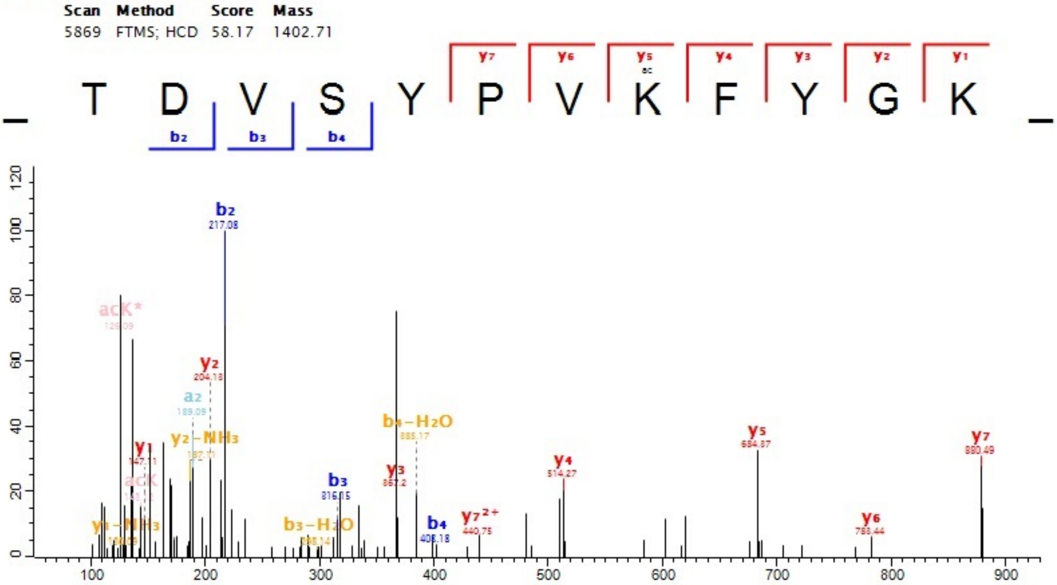


Q9AUV8-451:

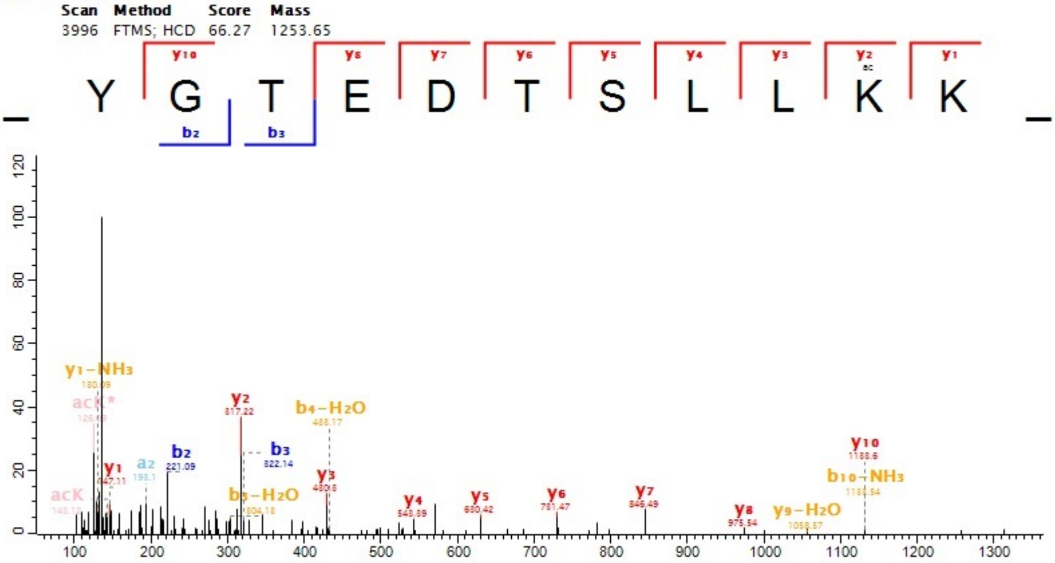

Q9AUV8-504:

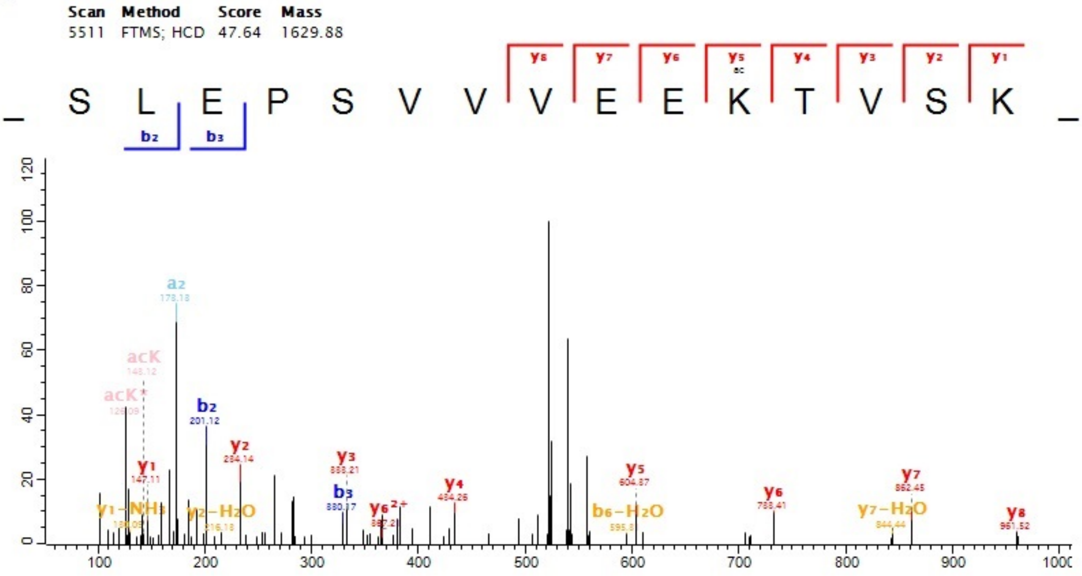


Q9AUV8-594:

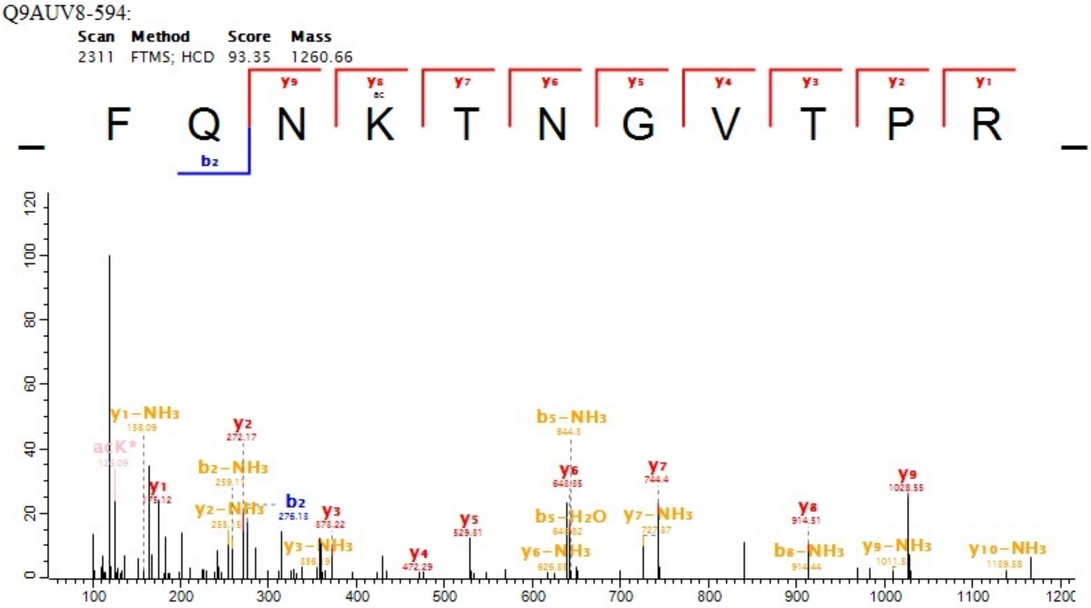


Q9AUV8-846:

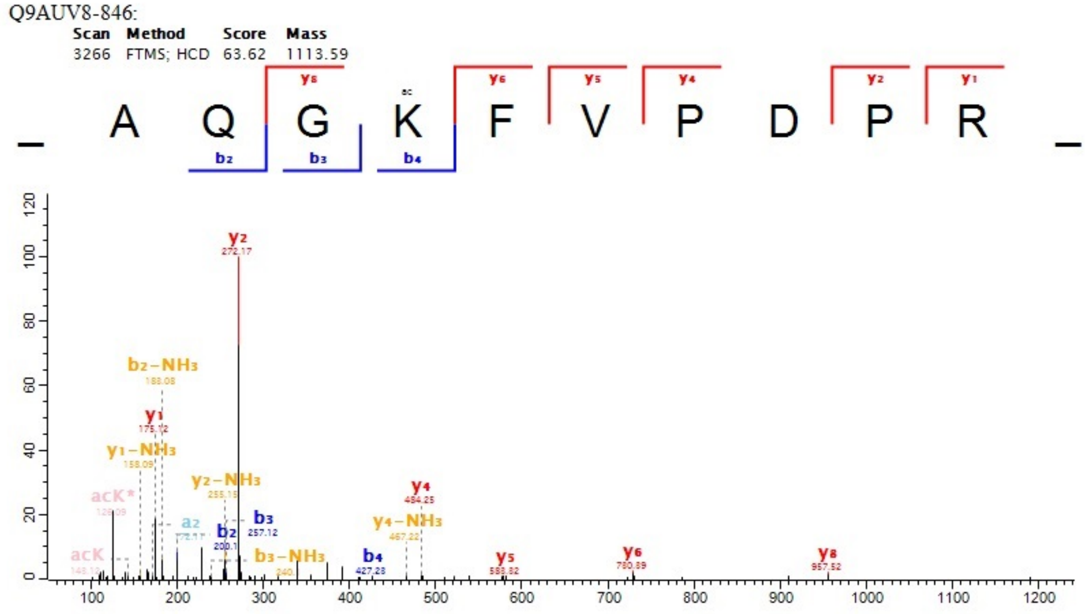

Q9AUV8-913:

| Scan | Method | Score | Mass |
|---|---|---|---|
| 3865 | FTMS; HCD | 122.74 | 945.5 |

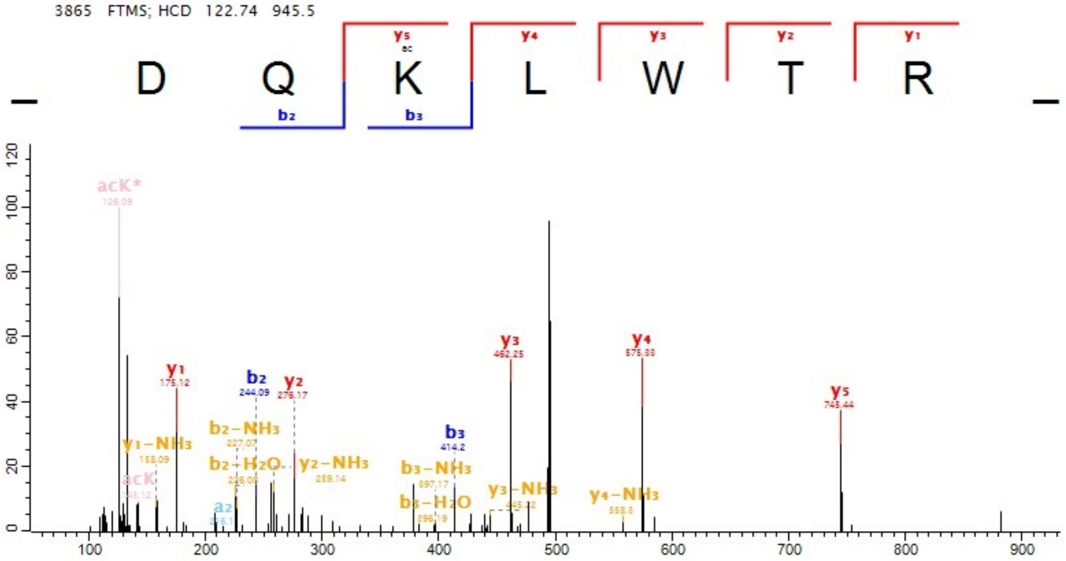


Q9AUV8-928:

| Scan | Method | Score | Mass |
|---|---|---|---|
| 5087 | FTMS; HCD | 96.21 | 1756.84 |

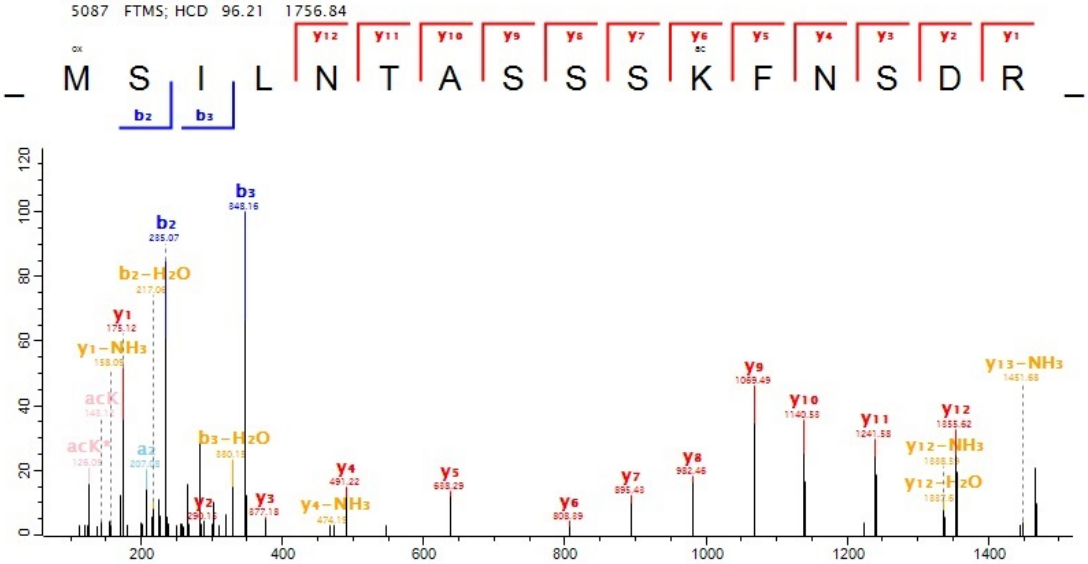

Supplement: S1 Fig — (PDF) [file pone.0168467.s001.pdf]
